# Risk of hematoma after hemithyroidectomy in an outpatient setting: a systematic review and meta-analysis

**DOI:** 10.1007/s00405-022-07312-y

**Published:** 2022-03-16

**Authors:** Karin Jeppesen, Caroline Moos, Tórhild Holm, Andreas Kristian Pedersen, Helene Skjøt-Arkil

**Affiliations:** 1 Department of Otorhinolaryngology, Head and Neck Surgery, University Hospital of Southern Denmark, Sønderborg, Denmark; 2 Department of Research and Learning, University Hospital of Southern Denmark, Aabenraa, Denmark; 3 Department of Regional Health Research, University Hospital of Southern Denmark, Aabenraa, Denmark; 4 Faculty of Health Science, University of Southern Denmark, Aabenraa, Denmark

**Keywords:** Hemithyroidectomy, Outpatient, Bayesian meta-analysis, Systematic review, Hematoma, Thyroidectomy

## Abstract

**Purpose:**

After thyroid surgery, the overriding concern is the risk of post-thyroid bleeding (PTB). This systematic review and meta-analysis aimed to assess the safety of hemithyroidectomy in an outpatient setting compared to an inpatient setting. The objectives were to (1) find the proportion of PTB in patients scheduled for outpatient hemithyroidectomy, (2) examine if outpatient hemithyroidectomy is clinically safe compared to an inpatient setting and (3) evaluate which selection criteria are most relevant for hemithyroidectomy in an outpatient setting.

**Methods:**

A systematic review was conducted using the following databases: MEDLINE (Ovid), EMBASE (Ovid) and the Cochrane Library from inception until September 2021. We included studies reporting on PTB of patients after hemithyroidectomy in an outpatient setting. The risk of bias was assessed using the Newcastle-Ottawa tool. The results were synthesised using Bayesian meta-analysis. Certainty in evidence was assessed using the GRADE approach.

**Results:**

This review included 11 cohort studies and 9 descriptive studies reporting solely on outpatients resulting in a total of 46,866 patients. PTB was experienced by 58 of the 9025 outpatients (0.6%) and 415 of the 37,841 inpatients (1.1%). There was no difference between the PTB rate of outpatients and inpatients (RR 0.715 CrI [0.396–1.243]). The certainty of the evidence was very low due to the high risk of bias.

**Conclusion:**

The risk of PTB in an outpatient setting is very low, and outpatient hemithyroidectomy should be considered clinically safe. The most relevant selection criteria to consider in outpatient hemithyroidectomy are (1) relevant comorbidities and (2) psycho/-social factors.

**Supplementary Information:**

The online version contains supplementary material available at 10.1007/s00405-022-07312-y.

## Introduction

Total thyroidectomy and hemithyroidectomy are commonly performed for benign thyroid disease but are also relevant in malignancy and are traditionally treated in an inpatient setting. However, outpatient procedures are increasing worldwide with advantages including faster recovery in accustomed surroundings and lower hospital expenses [1]. The first hemithyroidectomy in an outpatient setting was described from the US in 1986 [2]. Since then, outpatient thyroidectomies have increased [3], as has the diversity of opinions regarding safety in this setting. The primary concern of performing thyroidectomies in an outpatient setting is the development of post-thyroidectomy bleeding (PTB), which may lead to compression of the trachea followed by respiratory failure [4]. However, studies have shown that the risk of PTB for hemithyroidectomies compared to total thyroidectomies is low, and therefore many centres offer hemithyroidectomy in an outpatient setting [4–6]. A hemithyroidectomy leaves half of the thyroid gland and parathyroid glands undissected, a less invasive procedure with a lower risk of hypocalcemia [7] and bilateral recurrent laryngeal nerve injury. When assessing the risk of PTB, it is essential to distinguish between patient groups. Many studies reporting on outpatient thyroidectomy have not distinguished outcomes according to hemithyroidectomy versus total thyroidectomy [8–10].

To date, no systematic review has explicitly reported on the risk of PTB after a hemithyroidectomy in an outpatient setting.

Our hypothesis was that hemithyroidectomy in an outpatient setting is as safe as in an inpatient setting if selection criteria are appropriately applied. Therefore, the study aimed to assess the safety of hemithyroidectomy in an outpatient setting. The questions were:

1. What is the proportion of PTB in patients scheduled for an outpatient hemithyroidectomy?
2. Is outpatient hemithyroidectomy clinically safe compared to inpatient setting?
3. Which selection criteria are most relevant for hemithyroidectomy in an outpatient setting?

## Methods

Preferred Reporting Items for Systematic reviews and Meta-analyses (PRISMA) [11] and meta-analysis of observational studies in Epidemiology (MOOSE) guidelines [12] were used as the reporting guidelines for this systematic review. The study was registered in PROSPERO with the ID number 175568.

### Eligibility criteria

Publications were included if they: (a) reported outcomes from outpatient hemithyroidectomy, (b) reported on PTB, (c) described original research, and (d) were controlled studies (randomised trials, quasi-experimental studies, cohort studies) or descriptive studies. Descriptive studies were used to answer research question 1, whilst controlled studies were used to answer questions 2 and 3.

The exclusion criteria for studies included (a) patients who underwent bilateral thyroid surgery, completion thyroidectomy or complete cervical neck dissection, (b) conference abstracts and unpublished studies; (c) pregnant women or patients under 15 years of age; or (d) articles not in English or Danish.

We defined hemithyroidectomy as surgical removal of half of the thyroid gland or a part of the thyroid gland (lobectomy or isthmusectomy) in patients with benign or malignant thyroid disease. Surgery was performed under general anaesthesia using a cervical incision. The outpatient setting was defined as hospitalisation, surgery and discharge on the same calendar day. We defined PTB as local hematoma/wound/haemorrhage/re-bleeding after a hemithyroidectomy where intervention was required, such as re-surgery. However, hematomas requiring aspiration were recorded and included as a PTB. Seromas not requiring intervention were not considered a PTB.

### Search strategies

On 3rd April 2020, a comprehensive search of the literature from EMBASE (Ovid), MEDLINE (Ovid) and the Cochrane library was conducted after feedback from two university librarians specialising in medical research. We repeated this search on the 14th September 2021. The search algorithm was developed using keywords, synonyms and medical subject headings from the review question. The two key facets included the population (hemithyroidectomy) and the exposure (outpatients). There were no time restrictions to the search. The results of this structured search from MEDLINE (Ovid) and Embase (Ovid) are presented in Online Resource 1. In addition, we hand-searched the reference list of included studies and relevant systematic reviews.

### Selection process

The study selection process was performed using the screening tool Covidence (Veritas Health Innovation, Melbourne, Australia; www.covidence.org). The identified records were uploaded to Covidence for the removal of duplicates. The records were independently screened by two of three reviewers. The screening consisted of two steps—title/abstract screening and full-text screening. In title/abstract screening, one author (CM) screened all records, and the second independent screener was one of two authors (KJ or TH). One author (KJ) screened all records in full-text screening, and the second independent screener was one of two authors (CM or TH). Disagreements were resolved via consensus, with KJ having the deciding opinion. The full-text reports that did not meet the inclusion criteria were excluded according to specific reasons. Inter-rater agreement was quantified by Cohen’s kappa scores as well as percentage agreement [13]. We interpreted cut-offs for κ values as < 0.20 = poor, 0.21–0.40 = fair, 0.41–0.60 = moderate, 0.61–0.80 = good, and 0.81–1.00 = very good agreement.

### Data collection process

For each eligible study, information was collected by one reviewer (KJ) and checked independently by one of the other reviewers (TH, CM or HS). Disagreements were resolved via consensus, with KJ having the deciding opinion. Before data extraction began, three standardized forms were designed and trialled by KJ and TH: (1) characteristics of included studies, (2) selected outcome measures, (3) selection criteria for outpatient hemithyroidectomy. Then, the authors focussed on extracting data from the method, result and discussion sections of the included papers. The authors were not contacted if the information was missing or unclear but described in this study as ‘Not reported’.

The data extracted included: study design and setting (type, number of centres involved, and period of data collection), patient demographics (number of in- and outpatients, age, and sex), description of the surgeon (described skill by author, number of surgeons), the proportion of patients receiving drain, energy-based devices and/or topical biological adhesives (defined as adhesives used on the thyroid bed) postoperative protocol (outpatient observation period at hospital, and follow-up period), PTB incidence (number, proportion, and time of occurrence), infection, and recurrence of transient or permanent laryngeal nerve damage. In addition, the selection criteria for outpatient hemithyroidectomy including medical factors (age, ASA score, comorbidities, anticoagulation treatment, characteristics of pathology, and malignancy) and social factors (patient motivation with the postoperative setting, distance to hospital, sufficient understanding of the provided information, social/physical setting conducive to safe postoperative management, rapid access to a telephone, availability of a responsible and capable caregiver).

### Study risk of bias assessment

The overall quality was appraised using a tailored version of the Newcastle-Ottowa Scale (NOS) for cohort studies [14] whereby marital status was not considered a relevant confounder in the comparability domain and was therefore excluded. No eligible randomized studies were identified. Descriptive studies were not assessed. Two reviewers (CM and HS) independently assessed the studies, and any disagreements were resolved by discussion. The Newcastle-Ottowa assessment scale assesses studies according to (1) selection of exposed and non-exposed cohort, (2) comparability of the cohorts including controlling for confounders, and (3) outcome follow-up. A study can receive a maximum of four stars (one for selection, one for outcome and two for comparability). According to Agency for Health Research and Quality (AHRQ) standards, the number of stars can be converted to a description of the quality of the study: good, fair or poor.

### Measures of treatment effect

The primary outcome was PTB, and the effect measure used in the synthesis was risk ratio.

### Data analysis

The characteristics and results of the individual studies were presented in a tabular form. A quantitative synthesis (meta-analysis) was performed for the PTB outcome in the cohort studies comparing inpatients and outpatients using Bayesian statistics. If risk ratio and credibility intervals were not reported in a study, the reported raw data was used in the analysis. The credibility intervals for the risk ratio were calculated separately for each study. The choice of prior for the pooled estimates and each study risk ratio and other technical details can be seen in Online Resource 2.

The alpha level for this analysis was set at 0.05. Forest plots for Bayesian analysis were obtained using the computer programme Latex with the tikz package. Statistical heterogeneity was assessed using between-study variance [15], where the cut-offs were: no heterogeneity: τ = 0–0.1, low heterogeneity: τ = 0.1–0.5, high heterogeneity: τ = 0.5–1, extreme high heterogeneity: τ ≥ 1.

### Reporting bias assessment

Reporting bias was assessed by the construction of a funnel plot (Online Resource 3) to observe if asymmetry in the scatter of studies occurred.

### Certainty assessment

The quality of evidence for PTB was assessed using the Grades of Recommendations, Assessment, Development, and Evaluation (GRADE) approach [16]. The certainty of the evidence was classified as high, moderate, low, or very low. Observational studies start as low quality according to this approach. Two independent investigators conducted this process (CM and HS), and disagreements were solved by discussion.

## Results

### Study selection

**Fig. 1:**
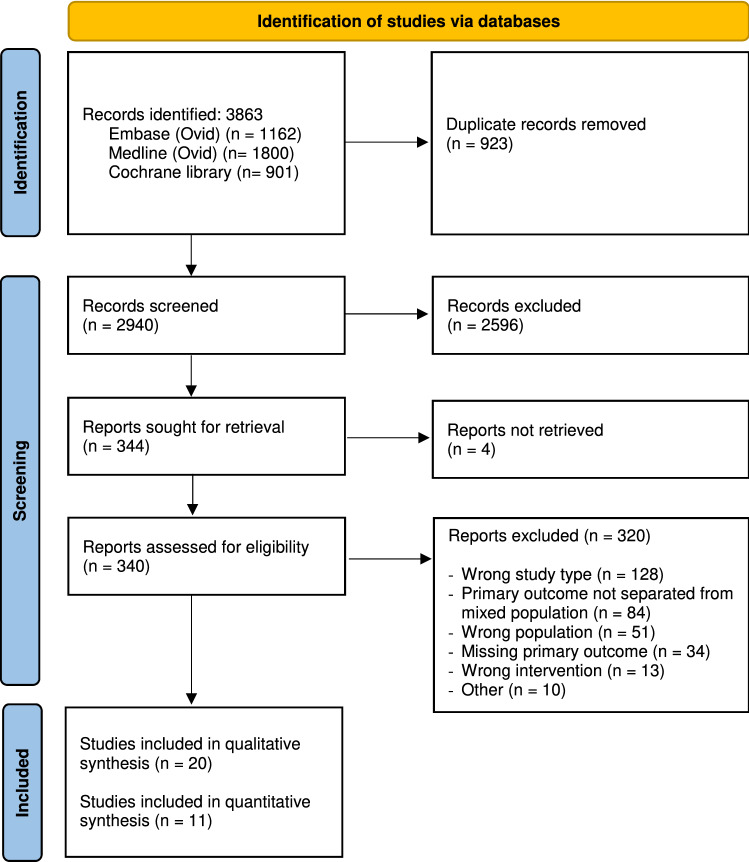
Flow Diagram

A total of 20 studies were included for qualitative synthesis in the review (see Fig. 1 Flow Diagram), eleven of these studies were included in a further quantitative synthesis (meta-analysis and bias evaluation).

The systematic literature search identified 3863 records (see Table 1). When duplicates were removed, 2940 records remained. After title and abstract screening, 340 full-text reports were assessed for eligibility, of which 320 reports did not meet the inclusion criteria, mainly because of wrong study type such as only conference abstract available. In addition, there were 84 studies excluded because PTB rate was not separated according to the population, e.g., if the population were mixed total and hemithyroidectomy. Furthermore, studies describing the outpatient setting as discharge < 23 h were excluded as the outpatient setting was defined as discharge within the same calendar day.

**Table 1:** Study Characteristics

Author, Year Country	Study design and setting - Study type - No of centres - Period of data collection	Type of patients - Inpatients: n - Outpatients: n	Age group - Inpatient: mean (range) - Outpatient: mean (range)	Sex distribution of female - Inpatient - Outpatient	Description of surgeon -Described skills by author - Number of surgeons	Postoperative protocol - Outpatient observation period at hospital - Follow-up period	Drain - Inpatients - Outpatients	Energy-based devices and/or topical biological adhesives
AlEssa, 2021 [34] Saudi Arabia	- Cohort study - Double - 2017–2019	- 3 - 17	- Not reported - Not reported	- Not reported - Not reported	- *Single faculty surgeon with 7 years of experience* - 1	- 8–10 h - 3 weeks	- Not reported - Not reported	Not reported
Almeida, 2010 [36] Portugal	- Cohort study - Single - 2005–2008	- 50 - 50	- 61* (47–71) - 47* (39–58)	- 72% - 80%	- Not reported - Not reported	- < 6 h - Not reported	- 96% - 86%	Not reported
Champault, 2009 [17] France	- Descriptive study - Single - 1999—2007	- Not relevant - 95	- Not relevant - 44 (16–46)^c^	- Not relevant - 84%	*- Surgeon with a high-volume sub specialty practice in neck and endocrine surgery* *-* 1	- 6 h - 3 weeks	- Not relevant - 0%	Bipolar coagulation
Chereau, 2021 [18] France	- Descriptive study - Single - 2018–2020	- Not relevant - 483	- Not relevant - Not reported	- Not relevant - Not reported	- Not reported - Not reported	- Not reported - Not reported	- Not relevant - 0%	Collagen pads
Chin, 2007 [19] Singapore	- Cohort study - Single - 2004–2005	- 64 - 50	- 51 (not reported) - 41 (not reported)	- 73% - 88%	*- Team with same consultant surgeon* - Not relevant	- < 6 h - 3 days	- 89% - 80%	Coagulation diathermy
de Boisanger, 2015 [20] Scotland	- Cohort study - Single - 2010–2013	- 35 - 145	- 48 (not reported) - 56 (not reported)	- 69% - 81%	*- Experienced surgeons* *-* 2	- Not reported - Not reported	- 20% - 1.4%	Not reported
Dulfer, 2016 [21] The Netherlands	- Cohort study - Double - 2015	- 12 - 31	- 61* (16–37) - 50* (14–86)	- 84% - 78%	*- Dedicated endocrine surgeons* *-* 2	- < 6 h - 2 weeks	- Not reported - Not reported	Not reported
Hessman, 2011 [22] USA	- Descriptive study - Single - 2004–2010	- Not relevant - 79	- Not relevant - Not reported	- Not relevant - Not reported	*- Single general surgeon* *-* 1	- > 2 h - 2 weeks	- Not relevant - 0%	Harmonic scalpel
Jeppesen, 2020 [23] Denmark	- Cohort study - Single - 2014–2019	- 92 - 137	- 57* (47–68) - 50* (41–55)	- 76% - 80%	*- Two experienced consultants* *-* 3	- 6 h - 3 months	- 4.3% - 0%	Bipolar forceps and Ligasure
Lacroix, 2014 [24] France	- Cohort study - Single - 2011–2012	- 112 - 34	- 53 (not reported) - 47^c^ (not reported)	- 76% - 81%	- Not reported - Not reported	- 6 h - 1 month	- Not reported - Not reported	Biological glue
Lo Gerfo, 1991 [25] USA	- Descriptive study - Single - 1987–1989	- Not relevant - 42	- Not relevant - Not reported	- Not relevant - Not relevant	- Not reported - Not reported	- 4–8 h - Not available	- Not relevant - 0%	Not reported
Mazeh, 2012 [26] USA	- Cohort study - Single - 2005–2011	- 92 - 279	- 60 (not reported) - 51 (not reported)	- 66% - 63%	- *Experienced endocrine surgeon* *-* 1	- 3–4 h - 30 days	- Not reported - Not reported^c^	Not reported
Noel, 2021 [35] Canada	- Cohort study - Multi (national based) - 1993–2017	- 37,353 - 6,794	- Not reported - Not reported	- Not reported - Not reported	- *Largely by high-volume surgeons* *-* Not reported	- Not reported - 30 days	- Not reported - Not reported	Not reported
Sklar, 2011 [27] Canada	- Descriptive study^a^ - Single - 2010–2011	- Not relevant - 81	- Not relevant - Not reported	- Not relevant - Not reported	- *Senior author* - Not reported	- Not reported - 7 days	- Not relevant - 0%	Not reported
Snyder, 2010 [28] USA	- Cohort study - Single - 2003–2009	- 21 - 340	- Not reported - Not reported	- Not reported - Not reported	- Not reported - 1	- Not reported - 30 days	- Not reported - 0%	Electrocautery for tiny vessels and synthetic collagen pads
Teoh, 2008 [29] Hong Kong	- Descriptive study - Single - 2005–2006	- Not relevant - 50^c^	- Not relevant - 45.6 (not reported)	- Not relevant - 88%	*- Surgical trainees under supervision of trained specialist* *-* Not reported	- Not reported - 4 weeks	- Not relevant - Not reported	Not reported
Terris, 2007 [30] USA	- Cohort study - Double - 2004–2005	- 7 - 35	- Not reported - Not reported	- Not reported - Not reported	*- Surgeon with a high volume practice* *-* 1	- Not reported - 1–2 weeks	- Not reported - Not relevant^c^	Not reported
Torfs, 2012 [31] Belgium	- Descriptive study - Single - 2008–2010	- Not relevant - 54	- Not relevant - 46 (27–67)^c^	- Not relevant - 81.5%	*- Surgeon assisted by resident* - 1	- 3 h - 6 weeks	- Not relevant - 0%	No devices used, only ligation
Trottier, 2009 [32] Canada	- Descriptive study - Single - 2002–2004	- Not relevant - 99	- Not relevant - Not reported	- Not relevant - Not reported	*- General surgeons with experience in the field but who did not have exclusive head and neck practice* - 3	- < 10 h - 2 weeks	- Not relevant - Not reported	Not reported
Yakhlef, 2017 [33] France	- Cohort study^b^ - Double - 2009–2013	- Not relevant - 130	- 50.5 (not reported) - 44 (not reported)	- 82% - 79%	*- Experienced endocrine surgeons* *- 3*	- Not available - 30 days	- Not reported - Not reported	Irrigated thermofusion or bipolar forceps

*Median

^a^Since the inpatient group included total and hemithyroidectomies we have chosen only to include the outpatient group. Trial period from 2009 to 2010 has not been included

^b^Although this is a cohort study in this review we have only used the outpatient group, as the inpatient group did not meet our inclusion criteria because of contralateral parathyroidectomy

^c^Part of exclusion criteria

Inter-rater agreement between CM and TH at the title/abstract screening was 85% (κ = 0.50,) and between CM and KJ 73% (κ = 0.85). At the full-text screening, the inter-rater agreement between KJ and CM was 84% (κ = 0.77) and 83% (κ = 0.36) between KJ and TH. Overall this corresponds to a ‘good’ agreement (mean κ = 0.62).

### Study characteristics

The included studies [17–36] comprised of 46,866 patients divided into 37,814 inpatients and 9025 outpatients. Study characteristics and data from each of these studies are presented in Table 1. Eleven of the included studies were cohort studies including an in- and outpatient group with 37,841 inpatients and 7912 outpatients, respectively [19–21, 23, 24, 26, 28, 30, 34–36]. Eight of the descriptive studies presented only the outpatient group consisting of 983 patients [17, 18, 22, 25, 27, 29, 31, 32]. One descriptive study was actually a cohort study with both an inpatient- and outpatient group, but as the inpatient group did not meet our inclusion criteria (included contralateral parathyroidectomy) we categorised the study as descriptive using only the outpatient group consisting of 130 patients [33]. Numerous studies have been published from various countries, with the largest originating from Canada [35], followed by the USA, France and Denmark [18, 23, 26, 28, 33]. Fifteen (75%) of the studies were from single-trial centres, and the data was collected between 1987 [25] until 2021 [35].

### Proportion of PTB in outpatients

In total, there were 473 of the 46,866 patients who experienced a PTB (1.0%) (Table 2). Of the inpatient group (37,841 patients) 415 experienced a PTB (1.1%) and of the outpatients (9025 patients) 58 had a PTB (0.6%). Most studies were observational studies with or without a comparator group from 1 to 2 centres describing the PTB cases in detail within a population. Noel’s study [35] was a national register study that reported PTB but did not describe the timing and treatment. The other studies reported 11 PTB cases, two required aspiration and nine patients needed re-surgery.

**Table 2:** Outcomes

Author, Year	PTB Incidence - Inpatient, *n *(%) - Outpatient, *n *(%)	Occurrence of PTB - Inpatient - Outpatient	Infection - Inpatients, *n *(%) - Outpatient, *n *(%)	Recurrent laryngeal nerve damage - Inpatient, *n *(%) - Outpatient, *n *(%)	
				Transient	Permanent
AlEssa, 2021 [34]	- 0 (0%) - 0 (0%)	- Not relevant - Not relevant	- Not reported - Not reported	- Not reported - Not reported	- Not reported - Not reported
Almeida, 2010 [36]	- 1 (2.0%) - 1 (2.0%)	- After discharge—surgical re-intervention - After discharge—surgical re-intervention	- 1 (2.0%) - 2 (4.0%)	- Not reported - Not reported	- 2 (4.0%) - 0 (0%)
Champault, 2009 [17]	- Not relevant - 1 (1.1%)	- Not relevant - < 6 h—surgical re-intervention^a^	- Not relevant - 0 (0%)	- Not relevant - 5 (5.3%)	- Not relevant - 0 (0%)
Chereau, 2021 [18]	- Not relevant - 1 (0.2%)	- Not relevant - 4 h—surgical re-intervention	- Not relevant - Not reported	- Not relevant - 8 (2%)	- Not relevant - Not reported
Chin, 2007 [19]	- 1 (1.6%) - 1 (2.0%)	- < 4 h—surgical re-intervention - < 4 h—surgical re-intervention	- 0 (0%) - 0 (0%)	- 5 (7.8%) - 2 (4.0%)	- 0 (0%) - 1 (1.6%)
de Boisanger, 2015 [20]	- 0 (0%) - 0 (0%)	- Not relevant - Not relevant	- 3 (8.6%) - 6 (4.1%)	- 0 (0%) - 0 (0%)	- 0 (0%) - 0 (0%)
Dulfer, 2016 [21]	- 0 (0%) - 0 (0%)	- Not relevant - Not relevant	- 0 (0%) - 0 (0%)	- 2 (16.7%) - 1 (3.2%)	- Not reported - Not reported
Hessman, 2011 [22]	- Not relevant - 1 (1.3%)	- Not relevant - 5 days, urgent decompression and surgical re-intervention^b^	- Not relevant - Not reported	- Not relevant - Not reported	- Not relevant - 0 (0%)
Jeppesen, 2020 [23]	- 0 (0%) - 0 (0%)	- Not relevant - Not relevant	- 1 (0.7%) - 2 (2.2%)	- 3 (2.2%) - 2 (2.2%)	- 1 (1.1%) - 2 (1.5%)
Lacroix, 2014 [24]	- 0 (0%) - 0 (0%)	- Not relevant - Not relevant	- 0 (0%) - 0 (0%)	- Not available - Not available	- 0 (0%) - 0 (0%)
Lo Gerfo, 1991 [25]	- Not relevant - 0 (0%)	- Not relevant - Not relevant	- Not relevant - 0 (0%)	- Not relevant - 0 (0%)	- Not relevant - Not reported
Mazeh, 2012 [26]	- 0 (0%) - 0 (0%)	- Not relevant - Not relevant	- Not reported - Not reported	-6 (2%) -2 (2%)	- 0 (0%) - 0 (0%)
Noel, 2021 [35]	- 413 (1.1%) - 49 (0.7%)	- Not reported - Not reported	- Not reported - Not reported	- Not reported - Not reported	- Not reported - Not reported
Sklar, 2011 [27]	- Not relevant - 0 (0%)	- Not relevant - Not relevant	- Not relevant - 0 (0%)	- Not relevant - Not reported	- Not relevant - Not reported
Snyder, 2010 [28]	- 0 (0%) - 1 (0.3%)	- Not relevant - 2 h—surgical re-intervention^a^	- Not reported - Not reported	- Not reported - Not reported	- Not reported - Not reported
Teoh, 2008 [29]	- Not relevant - 1 (2.0%)	- Not relevant - < 2 h—surgical re-intervention	- Not relevant - Not reported	- Not relevant - Not reported	- Not relevant - 1 (2.0%)
Terris, 2007 [30]	- 0 (0%) - 1 (2.9%)	- Not relevant - 8 days—aspiration	- Not reported - Not reported	- Not reported - Not reported	- 0 (0%) - 0 (0%)
Torfs, 2012 [31]	- Not relevant - 0 (0%)	- Not relevant - Not relevant	- Not relevant - 0 (0%)	- Not relevant - 0 (0%)	- Not relevant - 0 (0%)
Trottier, 2009 [32]	- Not relevant - 1 (1.0%)	- Not relevant - 2 days—aspiration	- Not relevant - 0 (0%)	- Not relevant - 0 (0%)	- Not relevant - 0 (0%)
Yakhlef, 2017 [33]	- Not relevant - 0 (0%)	- Not relevant - Not relevant	- Not reported - Not reported	- Not reported - Not reported	- Not reported - Not reported

^a^This patient took aspirin

^b^This patient had been restarted on warfarin postoperatively

In the included studies, the observation periods for outpatients varied between 2 and 6 h. Nine studies reported on timing of PTB [17–19, 22, 28–30, 32, 36]. Noel et al. [35] did not report on timing, despite having the largest study population of the included studies. The remaining ten studies had no PTB’s and therefore did not report on timing. Six of the nine patients experienced PTB (all required re-surgery) early within the observation period. The remaining three patients experienced PTB late on the 2nd, 5th or 8th day after surgery, one of them required re-operation while two required aspiration only.

### Clinical safety and meta-analysis

Eleven studies reported information on postoperative infection. In these studies, 1.4% of the patients in an inpatient setting had a postoperative infection (5/365 patients) compared to 1.2% of reported infections in outpatients (10/818 patients).

According to permanent recurrent laryngeal nerve damage, 12 studies reported inpatients risk of permanent laryngeal nerve damage as 0.7% (3/452 patients) and 0.4% for outpatients (4/1107 patients).

**Fig. 2:**
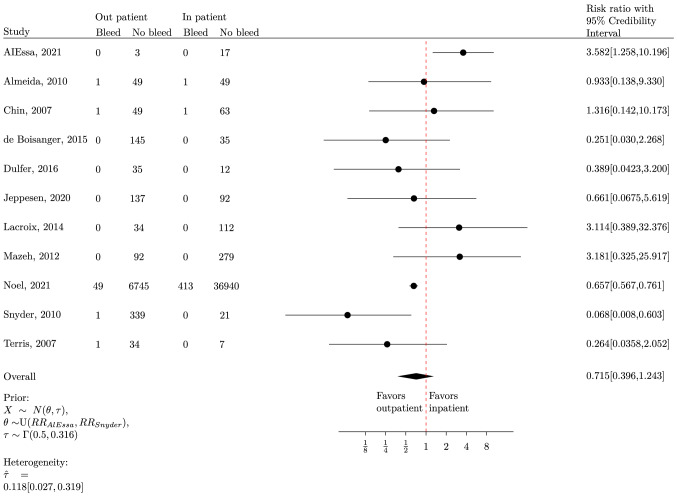
Forestplot

Eleven studies were included in the meta-analysis [19–21, 23, 24, 26, 28, 30, 34–36]. The result demonstrated a pooled RR of 0.715 (95% CrI 0.396–1.243), indicating no significant difference in PTB risk between in and outpatients but, leaning towards favouring outpatients (Fig. 2 Forestplot). This pooled summary showed low heterogeneity despite varying selection criteria indicated by a low tau of 0.118 (95% CI 0.027–0.319). The result of the sensitivity analysis can be found in Online Resource 4.

### Selection criteria

The heterogeneity between studies in selection criteria for outpatient hemithyroidectomy is considerable (see Table 3). The most common selection criteria for studies of outpatient hemithyroidectomy were ASA score, no relevant comorbidities, distance to the hospital and social/physical setting conducive to safe postoperative management. There was variation between ASA I-II or I-III in the selection criteria, but no studies considered ASA IV patients suitable for outpatient hemithyroidectomy. In addition, there was considerable variation between studies in relation to acceptable distance from the hospital.

**Table 3:**
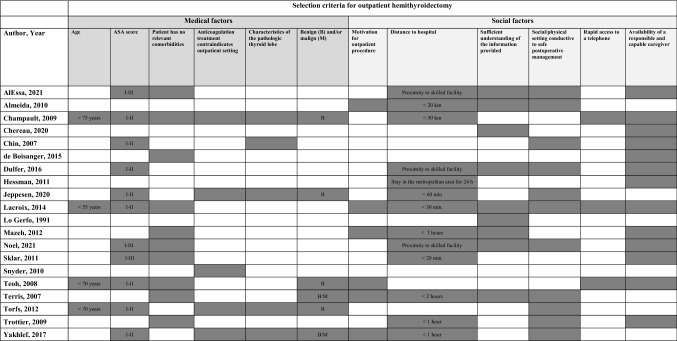
Selection criteria for outpatient hemithyroidectomy

### Study quality

In the risk of bias assessment (Online Resource 5) the studies scored relatively well for selection and outcome domains but low for comparability scale. None of the studies specifically reported risk ratio for hemithyroidectomy, and therefore all risk ratios were calculated from the raw frequency data in the published studies. Therefore we were unable to control for confounders. This has resulted in all studies being evaluated as poor quality.

A funnel plot was generated using the 11 cohort studies (Online Resource 3) which indicated no reporting bias.

The quality of evidence for PTB according to the GRADE system is presented in Online Resource 6. Observational studies are graded low, and the serious risk of bias downgraded the quality of evidence to be very low.

## Discussion

To our knowledge, this is the first systematic review and meta-analysis of the risk of PTB after hemithyroidectomy in an outpatient setting. We found nine descriptive studies reporting solely on outpatients and 11 cohort studies comprising a total of 46,866 patients (37,814 inpatients and 9025 outpatients). The proportion of PTB was low for outpatients confirming that hemithyroidectomy in an outpatient setting is safe. There were no significant differences between PTB risk for outpatients and inpatients (RR 0.715 CrI [0.396–1.243]), but the pooled RR does appear to lean towards favouring outpatients. This is not surprising given the selection criteria used to select patients with the lowest risk of bleeding for outpatient hemithyroidectomy. However, in general, we found a low risk of PTB regardless of in- or outpatient setting. The certainty of the evidence was very low due to the high risk of bias. Despite an apparent heterogeneity of the studies when selecting outpatients, the most helpful selection criteria appeared to be the status of relevant comorbidities (particularly bleeding disorders and anticoagulant treatment), home environment and social factors.

In 2017 Lee et al. [5] published a systematic review on thyroidectomy in an outpatient versus inpatient setting. Lee reported seven PTB cases from 1802 outpatients with a very low PTB risk of 0.4%. However, Lee’s research included total thyroidectomies. In addition, Lee conducted a meta-analysis on the overall complication rates and readmissions of hemithyroidectomy patients. Unfortunately, this meta-analysis only found two small studies for overall complications and three small studies for readmission/re-intervention rates, and Lee et al. did not separate PTB from overall complications. Therefore, the review from Lee et al. cannot be used to determine the specific risk of PTB after a hemithyroidectomy in an inpatient compared to an outpatient setting. However, Lee’s review does conclude that outpatient thyroidectomy is justified in a carefully selected patient group.

### Clinical considerations and definition of safety

The primary concern when performing thyroidectomies in an outpatient setting is the risk of PTB. In a worst-case scenario, PTB can lead to respiratory failure if unobserved or untreated [4]. Therefore PTB is a valuable outcome measure to assess if outpatient hemithyroidectomy is safe. Other complications affecting safety can be infections or recurrent nerve damage. Infections can also become a severe complication if not treated but usually develop more slowly, allowing time for the patient to contact a doctor to achieve antibiotic treatment. In hemithyroid surgery, nerve damage will only affect one nerve, and if the other recurrent nerve has a normal function, there will be no risk of serious respiratory failure in case of nerve damage. However, post-surgery patients can experience voice changes, and if the damage is persistent, speech therapy or other symptomatic treatment for a paralysed vocal cord may be necessary.

Fourteen of the 20 included studies in this review reported on recurrent nerve damage, some of which were transient and some permanent, making a general estimation of nerve damage difficult from this material. However, studies investigating nerve damage in thyroid surgeries report that exposure of the recurrent nerve and use of nerve monitoring reduced the rate of recurrent laryngeal nerve injury [37, 38]. When introducing day-care surgery, the patients must be well informed of the risks, particularly PTB, and infection and recurrent nerve damage should also be discussed. Centres hesitant in managing patients for thyroid surgery in an outpatient setting should consider patients requiring a hemithyroidectomy as a safe starting point.

### Surgeon skills

Generally, surgeons were experienced in the included studies, with approximately three surgeons per study. This information supports the general hypothesis that experienced surgeons perform more surgical procedures in outpatient settings [39], and therefore, the surgeon’s expertise could be considered a mediator. In future studies, we suggest that surgeon volume should be reported as the number of surgery cases per year rather than the current practice of simply reporting surgeon title [40, 41].

### Drainage

There seems to be a tendency to use fewer drains for thyroid surgery. Only two studies in this review [19, 36] used drains in more than 80% of their patients and these particular studies collected data between 2004 and 2008. Drainage is a possible confounding factor, but unfortunately could not be adjusted for in our meta-analysis because approximately half of the studies did not report information about it. However a systematic review and meta-analysis from 2017 investigating drainage after thyroid surgery reported no significant difference in PTB between patients in the drain versus no-drain group [42]. In future thyroid surgery studies, accurate reporting on drainage is essential to ensure confounding can be minimized.

### Energy-based devices and topical biological adhesives

Reporting of the use of energy-based devices and topical biological adhesives (haemostatic aids) is poor and only nine of the twenty studies report on the use of haemostatic aids. Furthermore, the reported devices and adhesives differ considerably. The available data in this review is not sufficient for an analysis that can determine whether there is a correlation between the use of haemostatic aids and PTB. Mahoney et al. reported that the use of energy devices for haemostasis during surgery was protective of PTB [43]. Therefore, energy-based devices and topical biological adhesives cannot be ruled out as confounding factors. Future studies should describe the operation-techniques in more detail to evaluate if different surgical techniques play a role in minimizing a patient’s risk of PTB.

### Observation after surgery

When considering outpatient surgery, the key question is the risk of PTB in the 6–24 h time period. Although some cases of PTB occur in this time interval, the majority of PTB’s occur within 6 h of surgery [40, 44]. Therefore, future feasibility studies for outpatient thyroid surgery must focus on a more accurate reporting of the timing of PTB complications.

### Selection criteria

Despite a lack of international guidelines for outpatient hemithyroidectomy, the American Thyroid Association has developed a guideline for selecting patients for outpatient thyroidectomy using a combination of the following factors: American Society of Anesthesiologists (ASA) score, patient understanding/capabilities, and home/geographical environment [45]. Many different studies have tried to identify factors associated with a higher PTB risk [4, 40, 42–44, 46]. Lang et al. found previous thyroid surgery and the size of the dominant nodule to be independent risk factors [44], whilst Godballe et al. identified age, male gender, malignant histology and extent of surgery (total thyroidectomy versus hemithyroidectomy) as independent risk factors for PTB [40]. Quimby et al. found Graves disease to be the only indication for patients at increased risk of PTB [46]. In 2021, Mahoney et al. reported that male gender, hypertension, diabetes and bleeding disorders were independent risk factors for PTB [43]. Another recent study from 2021 suggests that a risk score for PTB could be calculated based on two preoperative criteria; sex and anticoagulant treatment, to identify patients for which an outpatient setting is inappropriate [18]. The most relevant selection criteria for outpatient hemithyroidectomies were (1) status of relevant comorbidities (especially bleeding disorders and anticoagulant treatment) (2) social factors (including distance/time to hospital). Interestingly, despite heterogeneity of selection criteria there was still no difference in the risk of PTB according to setting.

### Study selection

In 2021 Sheikh et al. [47] investigated if outpatient hemithyroidectomy could be safely introduced in their centre. The study began with a literature-review reporting on experiences with outpatient hemithyroidectomy. In their literature-review they included a study from 2011 [48] with 782 outpatient hemithyroidectomies and a study from 2015 [49] describing outpatient thyroidectomy in 14,313 patients. Although these studies suggested outpatient hemithyroidectomy was safe in selected patients, these studies were ineligible for this review due to our in- and exclusion criteria; the last study included seromas not requiring intervention in the PTB rate and the first study did not report PTB as an independent complication. Nevertheless, both studies found low PTB-rates similar to those in this review.

### Study design and bias evaluation

The NOS for cohort studies was the risk of bias tool found most appropriate for the included studies. The risk of bias from all included studies is high because all studies scored poorly on the comparability domain. A study must be controlled for age, sex, marital status, or other named factors (maximum of two stars) to score highly in the comparability domain. However, if a study will control for confounders, the outcome needs to consist of at least one event. PTB is so rare in hemithyroidectomy patients that many studies reported zero events. Studies with outpatients groups, selected to minimize PTB risk, often reported zero or no PTB events. These controlled studies reported zero events but were unable to adjust for confounders because of this. The weighting of this particular domain in the NOS tool has resulted in most included studies being categorized as poor quality.

### Future considerations, strengths and limitations

Randomised controlled trials and large multicentre studies (e.g., population-based studies) are required to ensure the best evidence supports these results. Zhang et al. [50] recently published an RCT on outpatient total and hemithyroidectomy reporting that outpatient thyroidectomy is safe in patients with papillary thyroid carcinoma. This RCT was not relevant in this review as the outpatient setting described was discharge before 23 h. Completing an RCT that examines the risk of PTB in a selected outpatient population is challenging as the PTB outcome is so rare. A considerable number of patients would be required in the study to ensure the outcome could be registered.

The strength of our study is the use of Bayesian statistics enabling us to manage studies reporting zero events as outcomes and the inclusion of one very large study. The use of the Bayesian statistic also gives an estimate of the true risk ratio. However, this study’s greatest limitation is the calculation of RR from raw frequency data reported in the individual studies. However, although RR could be calculated, it was not possible to adjust for confounders. Another limitation is that this review captures studies that report on an extremely infrequent event. These studies are relevant since they report on the very low risk of PTB after a hemithyroidectomy but the PTB event is so infrequent that 10 of the included studies did not capture the outcome in the population, making statistical analysis complicated.

## Conclusion

In this first review investigating the risk of PTB after outpatient hemithyroidectomies, we found that the risk of PTB in an outpatient setting is very low, and outpatient hemithyroidectomy can be considered clinically safe. In addition, the most relevant selection criteria for clinically safe outpatient hemithyroidectomy to consider are (1) relevant comorbidities and (2) psycho/-social factors.

## Supplementary Information

Below is the link to the electronic supplementary material.

Supplementary file1 (PDF 121 KB)
