# Supplementary material for: Risk of hematoma after hemithyroidectomy in an outpatient setting: a systematic review and meta-analysis
Source: Eur Arch Otorhinolaryngol. 2022 Mar 16;279(8):3755–67. doi: 10.1007/s00405-022-07312-y (PMC9249722; doi:10.1007/s00405-022-07312-y)
Supplement: Supplementary file 1 — Supplementary file1 (PDF 121 KB) [file 405_2022_7312_MOESM1_ESM.pdf]

Risk of hematoma after hemithyroidectomy in an outpatient setting a systematic review and meta-analysis

European Archives of Oto-Rhino-Laryngology

Karin Jeppesen[a*], Caroline Moos[b,c], Tórhild Holm[d], Andreas Kristian Pedersen[b,c] & Helene Skjøt-Arkil[b,c]

a) Department of Otorhinolaryngology, Head and Neck Surgery, University Hospital of Southern Denmark
b) Department of Research and Learning, University Hospital of Southern Denmark
c) Department of Regional Health Research University Hospital of Southern Denmark
d) Faculty of Health Science, University of Southern Denmark

- Corresponding author: Karin Jeppesen MD, mail: karin.jeppesen@rsyd.dk

Online Resource 1a Search strategy from Embase via Ovid

Set number	Term searched or sets combined
#1	thyroidectomy/ or hemithyroidectomy.mp.
#2	thyroidectomy/ or subtotal thyroidectomy/ or "patient history of partial thyroidectomy"/ or thyroidectomy.mp.
#3	isthmusectomy.mp
#4	thyroid surgery.mp.
#5	1 or 2 or 3 or 4
#6	ambulatory surgery/ or day-case.mp.
#7	ambulatory surgery/ or day case.mp
#8	day surgery.mp. or ambulatory surgery/
#9	day surgery.mp.
#10	day care.mp.
#11	day-care.mp.
#12	day-stay.mp.
#13	(Length of Stay or Ambulatory Surgical Procedures or Patient Discharge).mp.
#14	day patient.mp.
#15	ambulant.mp.
#16	(Ambulatory Care or ambulatory or Ambulatory Surgical Procedures).mp.
#17	outpatient care/ or outpatient.mp. or outpatient/ or outpatient department/
#18	out patient.mp.
#19	out-patient.mp.
#20	(partial adj3 (hospitalization or hospitalizations or hospitalisation or hospitalizations)).mp.
#21	day care.mp. or day care/
#22	ambulatory care.mp. or ambulatory care/
#23	same day.mp.
#24	short stay.mp.
#25	Ambulatory Surgery.mp. or ambulatory surgery/
#26	6 or 7 or 8 or 9 or 10 or 11 or 12 or 13 or 14 or 15 or 16 or 17 or 18 or 19 or 20 or 21 or 22 or 23 or 24 or 25
#27	5 and 26
#28	limit 27 to english language

Online Resource 1b Search strategy from Medline via Ovid

Search number	Search terms
#1	Thyroidectomy/ or Thyroid Gland/ or Hemithyroidectomy.mp. or Thyroid Diseases/
#2	thyroidectomy.mp. or Thyroidectomy
#3	isthmusectomy.mp.
#4	thyroid surgery.mp.
#5	1 or 2 or 3 or 4
#6	Ambulatory Surgical Procedures/ or day-case.mp
#7	Ambulatory Surgical Procedures/ or day case.mp.
#8	day surgery.mp. or Ambulatory Surgical Procedures/
#9	day-surgery.mp.
#10	day care.mp.
#11	day-care.mp.
#12	day-stay.mp.
#13	"Length of Stay"/ or Ambulatory Surgical Procedures/ or Patient Discharge/
#14	day patient.mp.
#15	ambulant.mp
#16	Ambulatory Care/ or ambulatory.mp. or Ambulatory Surgical Procedures/
#17	outpatient.mp. or Outpatients/
#18	Out patient.mp.
#19	out-patient.mp.
#20	(partial adj3 (hospitalization or hospitalizations or hospitalisation or hospitalizations)).mp.
#21	day care.mp. or Day Care, Medical/
#22	ambulatory care.mp. or Ambulatory Care/
#23	same day.mp
#24	short stay.mp.
#25	6 or 7 or 8 or 9 or 10 or 11 or 12 or 13 or 14 or 15 or 16 or 17 or 18 or 19 or 20 or 21 or 22 or 23 or 24
#26	5 and 25
#27	limit 26 to english language

Online Resource 1c Search strategy from Cochrane library

#1	Hemithyroidectomy or thyroidectomy or isthmusectomy or Thyroid surgery
#2	Day case or day-case or day surgery or day-surgery or day care or day-care or day stay or day-stay or day patient or ambulant or ambulatory or out patient or outpatient or out-patient or hospitalization or hospitalisation or hospitalizations or hospitalisations or Ambulatory surgical procedures or Ambulatory Care or Same day or Short stay or ambulatory surgery
#3	#1 and #2

Risk of hematoma after hemithyroidectomy in an outpatient setting a systematic review and meta-analysis

European Archives of Oto-Rhino-Laryngology

Karin Jeppesen[a*], Caroline Moos[b,c], Tórhild Holm[d], Andreas Kristian Pedersen[b,c] & Helene Skjøt-Arkil[b,c]

a) Department of Otorhinolaryngology, Head and Neck Surgery, University Hospital of Southern Denmark
b) Department of Research and Learning, University Hospital of Southern Denmark
c) Department of Regional Health Research University Hospital of Southern Denmark
d) Faculty of Health Science, University of Southern Denmark

- Corresponding author: Karin Jeppesen MD, mail: karin.jeppesen@rsyd.dk

Online Resource 2 Extended Data Analysis

A quantitative synthesis (meta-analysis) was performed for the PTB outcome in the cohort studies comparing inpatients and outpatients using a Bayesian statistics. We choose Bayesian statistics as several of the studies reported no bleeders and the inference drawn from the meta analysis favors the Bayesian approach. If risk ratio and credibility intervals were not reported in a study, the reported raw data was used in the analysis. The credibility intervals for the risk ratio were calculated separately for each study by way of the methods described in Möller et al (1).

The prior used for the calculation was assumed to be beta-distributed where both shape parameters were equivalent to one (1). In order to estimate the pooled risk ratio and its corresponding 95% credibility interval a Bayesian linear regression was utilized on the log risk ratio. The prior for this model was set to follow a normal distribution where the mean and the standard deviation followed different priors.

The means prior followed a uniform distribution with support from a to b, where a was set to be the smallest of log risk ratios of the included studies and b to be the largest log risk ratio of the included studies, and the standard deviation followed a prior, which was a gamma distribution with shape parameter 0.5 and scale parameter 0.316, such that diagnostics of the model were satisfactory. We choose a flat prior for the log risk ratio as the flat prior is uninformative and therefore follows the guidelines proposed by (2). To explore how acceptable the priors for the linear regression were, diagnostic plots consisting of trace, auto correlation and density plots were conducted.

Statistical heterogeneity was assessed using between-study variance (3), where the cut-offs were: no heterogeneity: τ=0-0.1, low heterogeneity: τ=0.1-0.5, high heterogeneity: τ=0.5-1, extreme high heterogeneity: τ=>1. Note however, the statistical heterogeneity is strongly influenced by the choice of priors, and should be interpreted with caution.

To assess publication bias a funnel plot was conducted. We choose not to draw the funnel itself as the precision of the given estimates is affected of the priors.

A sensitivity analysis was conducted where we choose a different prior for the mean. Instead of using Alessa risk ratio we choosed to set b to the risk ratio estimated in Mazeh. The choice of priors can also be seen in the forest plots.

The alpha level for this analysis was set at 0.05. Forest plots for Bayesian analysis were obtained using the computer program Latex with the tikz package and the analysis itself was done in STATA by way of the bayesmh module both for calculating the credibility interval for the individual studies and for the pooled risk ratio.

Reference list

1. Möller S, Ahrenfeldt LJ. Estimating Relative Risk When Observing Zero Events-Frequentist Inference and Bayesian Credibility Intervals. Int J Environ Res Public Health. 2021;18(11).
2. Dias S, Welton NJ, Sutton AJ, Ades AE. NICE Decision Support Unit Technical Support Documents. NICE DSU Technical Support Document 2: A Generalised Linear Modelling Framework for Pairwise and Network Meta-Analysis of Randomised Controlled Trials. London: National Institute for Health and Care Excellence (NICE)

Copyright © 2014 National Institute for Health and Clinical Excellence, unless otherwise stated. All rights reserved.; 2014.
3. Turner RM, Jackson D, Wei Y, Thompson SG, Higgins JP. Predictive distributions for between-study heterogeneity and simple methods for their application in Bayesian meta-analysis. Stat Med. 2015;34(6):984-98.

Risk of hematoma after hemithyroidectomy in an outpatient setting a systematic review and meta-analysis

European Archives of Oto-Rhino-Laryngology

Karin Jeppesen[a*], Caroline Moos[b,c], Tórhild Holm[d], Andreas Kristian Pedersen[b,c] & Helene Skjøt-Arkil[b,c]

a) Department of Otorhinolaryngology, Head and Neck Surgery, University Hospital of Southern Denmark
b) Department of Research and Learning, University Hospital of Southern Denmark
c) Department of Regional Health Research University Hospital of Southern Denmark
d) Faculty of Health Science, University of Southern Denmark

- Corresponding author: Karin Jeppesen MD, mail: karin.jeppesen@rsyd.dk

Funnelplot

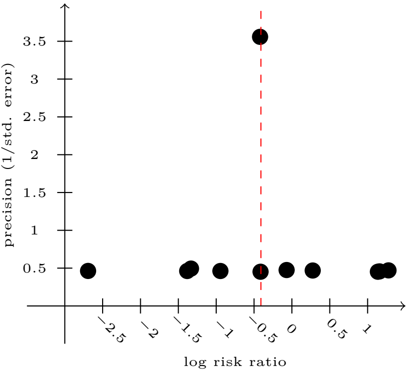

precision (1/std. error)
3.5
3
2.5
2
1.5
1
0.5
−2.5
−2
−1.5
−1
−0.5
0
0.5
1
log risk ratio

Risk of hematoma after hemithyroidectomy in an outpatient setting a systematic review and meta-analysis

European Archives of Oto-Rhino-Laryngology

Karin Jeppesen[a*], Caroline Moos[b,c], Tórhild Holm[d], Andreas Kristian Pedersen[b,c] & Helene Skjøt-Arkil[b,c]

a) Department of Otorhinolaryngology, Head and Neck Surgery, University Hospital of Southern Denmark
b) Department of Research and Learning, University Hospital of Southern Denmark
c) Department of Regional Health Research University Hospital of Southern Denmark
d) Faculty of Health Science, University of Southern Denmark

- Corresponding author: Karin Jeppesen MD, mail: karin.jeppesen@rsyd.dk

Online Resource 4 Results of sensitivity analysis

The sensitivity analysis showed a significant difference between the two groups (risk ratio 0.641; 95% CrI 0.453-0.903), and a high to an extremely high degree of heterogeneity (τ=0.494; 95% CrI 0.166-1.012).

Risk of hematoma after hemithyroidectomy in an outpatient setting a systematic review and meta-analysis

European Archives of Oto-Rhino-Laryngology

Karin Jeppesen[a*], Caroline Moos[b,c], Tórhild Holm[d], Andreas Kristian Pedersen[b,c] & Helene Skjøt-Arkil[b,c]

a) Department of Otorhinolaryngology, Head and Neck Surgery, University Hospital of Southern Denmark
b) Department of Research and Learning, University Hospital of Southern Denmark
c) Department of Regional Health Research University Hospital of Southern Denmark
d) Faculty of Health Science, University of Southern Denmark

- Corresponding author: Karin Jeppesen MD, mail: karin.jeppesen@rsyd.dk

Online Resource 5 Bias evaluation based on Newcastle-Ottowa Scale for cohort studies

	**Selection**				**Comparability**		**Outcome**			**Agency for Health Research and Quality (AHRQ) standard**
**Author, Year**	Representativeness of the exposed cohort	Selection of the non-exposed cohort	Ascertainment of exposure	Demonstration that outcome of interest was not present at start of study	Study controls for age and sex	Study controls for other factors	Assessment of outcome	Was follow-up long enough for outcomes to occur	Adequacy of follow up	Good = 3-4 * selection + 1-2 * comparability + 2-3 * outcome<br>Fair = 2 * selection + 1-2* comparability + 2-3 * outcome<br>Poor = 0-1 * selection + 0 * comparability + 1 * outcome
**AlEssa, 2021**	*	*	*	*	-	-	*	*	*	Poor
**Almeida, 2010**	*	*	*	*	-	-	*	-	-	Poor
**Chin, 2007**	*	*	*	*	-	-	*	-	*	Poor
**de Boisanger, 2015**	*	*	*	*	- [a]	- [a]	*	-	-	Poor
**Dulfer, 2016**	*	*	*	*	- [a]	- [a]	*	*	*	Poor
**Jeppesen, 2020**	*	*	*	*	- [a]	- [a]	*	*	*	Poor
**Lacroix, 2014**	*	*	*	*	- [a]	- [a]	*	*	*	Poor
**Mazeh, 2012**	-	*	*	*	- [a]	- [a]	*	*	*	Poor
**Noel, 2021**	*	*	*	*	-	-	*	*	*	Poor
**Snyder, 2010**	*	*	*	*	-	-	*	*	*	Poor
**Terris, 2007**	*	*	*	*	-	-	*	*	*	Poor

[a] These studies reported no PTB outcomes and therefore it was not possible to control for different variables.

Risk of hematoma after hemithyroidectomy in an outpatient setting a systematic review and meta-analysis

European Archives of Oto-Rhino-Laryngology

Karin Jeppesen[a*], Caroline Moos[b,c], Tórhild Holm[d], Andreas Kristian Pedersen[b,c] & Helene Skjøt-Arkil[b,c]

a) Department of Otorhinolaryngology, Head and Neck Surgery, University Hospital of Southern Denmark
b) Department of Research and Learning, University Hospital of Southern Denmark
c) Department of Regional Health Research University Hospital of Southern Denmark
d) Faculty of Health Science, University of Southern Denmark

- Corresponding author: Karin Jeppesen MD, mail: karin.jeppesen@rsyd.dk

Online Resource 6 GRADE evidence profile of PTB

Outcome	Study design	Risk of bias	Inconsistency	Indirectness	Imprecision	Publication bias	Other considerations	Quality
PTB	Observational	Serious	Low heterogeneity	No serious indirectness	No serious imprecision	No serious publication bias	None	* Very low
